# Applications of machine learning to undifferentiated chest pain in the emergency department: A systematic review

**DOI:** 10.1371/journal.pone.0252612

**Published:** 2021-08-24

**Authors:** Jonathon Stewart, Juan Lu, Adrian Goudie, Mohammed Bennamoun, Peter Sprivulis, Frank Sanfillipo, Girish Dwivedi

**Affiliations:** 1 School of Medicine, The University of Western Australia, Crawley, Western Australia, Australia; 2 Harry Perkins Institute of Medical Research, Murdoch, Western Australia, Australia; 3 School of Physics, Mathematics and Computing, University of Western Australia, Crawley, Western Australia, Australia; 4 Department of Emergency Medicine, Fiona Stanley Hospital, Murdoch, Western Australia, Australia; 5 Department of Health Western Australia, East Perth, Western Australia, Australia; 6 School of Population and Global Health, University of Western Australia, Crawley, Western Australia, Australia; 7 Department of Cardiology, Fiona Stanley Hospital, Murdoch, Western Australia, Australia; University of Palermo, ITALY

## Abstract

**Background:**

Chest pain is amongst the most common reason for presentation to the emergency department (ED). There are many causes of chest pain, and it is important for the emergency physician to quickly and accurately diagnose life threatening causes such as acute myocardial infarction (AMI). Multiple clinical decision tools have been developed to assist clinicians in risk stratifying patients with chest. There is growing recognition that machine learning (ML) will have a significant impact on the practice of medicine in the near future and may assist with diagnosis and risk stratification. This systematic review aims to evaluate how ML has been applied to adults presenting to the ED with undifferentiated chest pain and assess if ML models show improved performance when compared to physicians or current risk stratification techniques.

**Methods and findings:**

We conducted a systematic review of journal articles that applied a ML technique to an adult patient presenting to an emergency department with undifferentiated chest pain. Multiple databases were searched from inception through to November 2020. In total, 3361 articles were screened, and 23 articles were included. We did not conduct a metanalysis due to a high level of heterogeneity between studies in both their methods, and reporting. The most common primary outcomes assessed were diagnosis of acute myocardial infarction (AMI) (12 studies), and prognosis of major adverse cardiovascular event (MACE) (6 studies). There were 14 retrospective studies and 5 prospective studies. Four studies reported the development of a machine learning model retrospectively then tested it prospectively. The most common machine learning methods used were artificial neural networks (14 studies), random forest (6 studies), support vector machine (5 studies), and gradient boosting (2 studies). Multiple studies achieved high accuracy in both the diagnosis of AMI in the ED setting, and in predicting mortality and composite outcomes over various timeframes. ML outperformed existing risk stratification scores in all cases, and physicians in three out of four cases. The majority of studies were single centre, retrospective, and without prospective or external validation. There were only 3 studies that were considered low risk of bias and had low applicability concerns. Two studies reported integrating the ML model into clinical practice.

**Conclusions:**

Research on applications of ML for undifferentiated chest pain in the ED has been ongoing for decades. ML has been reported to outperform emergency physicians and current risk stratification tools to diagnose AMI and predict MACE but has rarely been integrated into practice. Many studies assessing the use of ML in undifferentiated chest pain in the ED have a high risk of bias. It is important that future studies make use of recently developed standardised ML reporting guidelines, register their protocols, and share their datasets and code. Future work is required to assess the impact of ML model implementation on clinical decision making, patient orientated outcomes, and patient and physician acceptability.

**Trial registration:**

International Prospective Register of Systematic Reviews registration number: CRD42020184977.

## Introduction

Complex decision-making amongst uncertainty is at the core of emergency medicine [1]. Emergency physicians must manage parallel and competing demands in an often chaotic and unpredictable environment. There is an ongoing challenge in identifying patients with potentially life-threatening conditions from more common benign diagnosis. Chest pain exemplifies this diagnostic challenge.

Chest pain is amongst the most common reason for presentation to the emergency department (ED) [2]. There are many causes of chest pain, and it is important for the emergency physician to quickly and accurately assess, investigate, and diagnose life threatening causes such as acute coronary syndrome (ACS). ACS encompasses a range of important diagnosis related to cardiac ischemia including unstable angina (UA), non-ST elevation myocardial infarction (NSTEMI), and ST elevation myocardial infarction (STEMI) [3]. ACS causes significant mortality and morbidity, and outcomes are improved with early recognition and treatment [4].

The majority of patients who present to an ED with chest pain will not have ACS [5]. Risk stratification is an integral part of the evaluation of chest pain [6]. History and physical examination alone are unreliable in evaluating patients with chest pain [7]. This has led to the development of multiple clinical decision tools such as the TIMI score and the HEART score to assist clinicians in determining which patients with chest pain are at high risk of acute coronary syndrome [8, 9]. Many of these decision tools have been validated internationally in multiple prospective trials and the HEART score has achieved good results [10]. Despite these decision tools, a small number of cases of ACS are still missed [11] There is growing recognition that emerging artificial intelligence (AI) technologies will have a significant impact on the practice of medicine in the near future [12, 13]. There has been longstanding interest in the application of AI based techniques to chest pain [14].

The field of artificial intelligence can be broadly and pragmatically defined as “the theory and development of computer systems able to perform tasks normally requiring human intelligence” [15]. Over the last decade a combination of exponential increases in computing power, the digitalisation of data, and advances in AI algorithms has led to a renaissance in AI research [16]. Machine learning (ML) is a subfield of AI that uses various methods to automatically detect patterns in data, then use these patterns to make predictions or decisions [17]. By repeatedly comparing predictions with results, machine learning models iteratively adjust their internal parameters (a process called “training”) to improve their performance. A trained model’s predictions can then be tested on unseen data to ensure that the model can be generalised to new data and that it has not become ‘over fit’ to the data that was used to train it. Deep learning (DL) is a type of ML that uses a large number of interconnected non-linear processing units to obtain increasingly abstract representations of data, giving it the capability to learn to model very complex functions [18]. DL algorithms have been used to achieve impressive results in multiple diverse fields such as image recognition, speech recognition, and natural language processing [19–22].

State of the art ML technologies are overwhelmingly narrow rather than general in their current applications but have still achieved great successes, including on some problems previously thought to be intractable [23]. There are ongoing efforts to create more generalisable models, however application of already existing narrow ML technologies could still fundamentally change many industries including healthcare [24]. AI techniques have demonstrated capability to predict patient outcomes and risk stratify patients based on clinical and physiological data [25, 26]. AI techniques have recently been applied with success to the diagnosis of myocardial infarction [27]. The implementation of artificial intelligence techniques into clinical practice remains a challenge.

This systematic review aims to evaluate the applications of machine learning in undifferentiated chest pain in the ED by answering the following questions.

1. How has ML been applied to adults presenting to the ED with undifferentiated chest pain?
2. Do ML models show improved performance compared to physicians or current risk stratification techniques?

## Methods

A systematic review protocol was reprepared in accordance with PRISMA-P guidelines and registered with the International Prospective Register of Systematic Reviews (PROSPERO) on 08/09/2020 (registration number CRD42020184977) [28]. We conducted and report this review in line with the PRISMA Reporting Guidelines for Systematic Reviews [29].

We included all journal articles that applied a ML technique to an adult patient presenting to an ED with undifferentiated chest pain. As this study aims to broadly assess the capability of ML applied to undifferentiated chest pain in the ED, all outcomes and comparators were included. Studies that did not use a comparator were still included in our review. We excluded conference abstracts, studies that did not use ML techniques, studies that did not assess undifferentiated chest pain, studies not based in an ED setting, and studies that focused solely on using ML for imaging or investigation interpretation.

The search strategy for this systematic review was developed with input from study authors and a health sciences librarian with expertise in systematic review searching. We searched Pubmed (MEDLINE), Cochrane Library, Web of Science, Embase, and Scopus for English language articles published from database inception to 11/08/2020. Electronic databases were first searched on 11/08/2020 and last searched on 15/11/2020. We searched for medical subject headings (MeSH) words and text keywords related to chest pain, artificial intelligence, machine learning, deep learning, and emergency medicine. The MEDLINE search strategy is provided in S1 Appendix. The MEDLINE strategy was adapted to the other databases. Reference lists of all included studies and authors personal archives were also reviewed for further relevant literature to ensure literature saturation was achieved.

Citations and abstracts were screened by two reviewers (JS and JL) against predefined inclusion and exclusion criteria. Both of the review authors were blind to the journal titles, study authors, and institutions. Full text articles were obtained for any articles identified by one reviewer to meet criteria. Two reviewers (JS and JL) then screened the full text reports against inclusion and exclusion criteria. Data were extracted by JS and JL using a standardised form. The form was piloted, and calibration exercises were conducted prior to formal data extraction to ensure consistency between reviewers. In all cases of conflict or discrepancy, additional study authors were involved until a decision was reached. Study authors were contacted by email to resolve any significant uncertainties.

Data extracted included study type, outcomes, population, input data used, ML methodology used, number of input variables in the ML model, comparisons, results, public availability of dataset, and public availability of model code. Risk of bias in studies was assessed by two authors (JS and JL) and using the Prediction model Risk of Bias Assessment Tool (PROBAST) [30].

We did not conduct a metanalysis due to a high level of heterogeneity between studies in both their methods, and reporting. We conducted a narrative analysis of the included studies to provide further commentary and exploration of the trends and findings.

## Results

### Study selection

We identified 3590 records following database searching and a further 42 records through other sources, including authors personal libraries. Following removal of duplicates, 3361 records remained and underwent title and abstract screening. 3279 records were excluded. The remaining 82 full-text articles were assessed for eligibility.

In total, 59 articles were excluded for the following reasons:

13 articles were excluded as they were an abstract only.
11 articles were excluded as they were a commentary or review
1 article was excluded as it focused only on CTCA result interpretation
15 articles were excluded as they did not use ML.
17 articles were excluded as they focused only on ECG interpretation.
2 articles were excluded as they did not focus on undifferentiated chest pain in the ED

**Fig 1:**
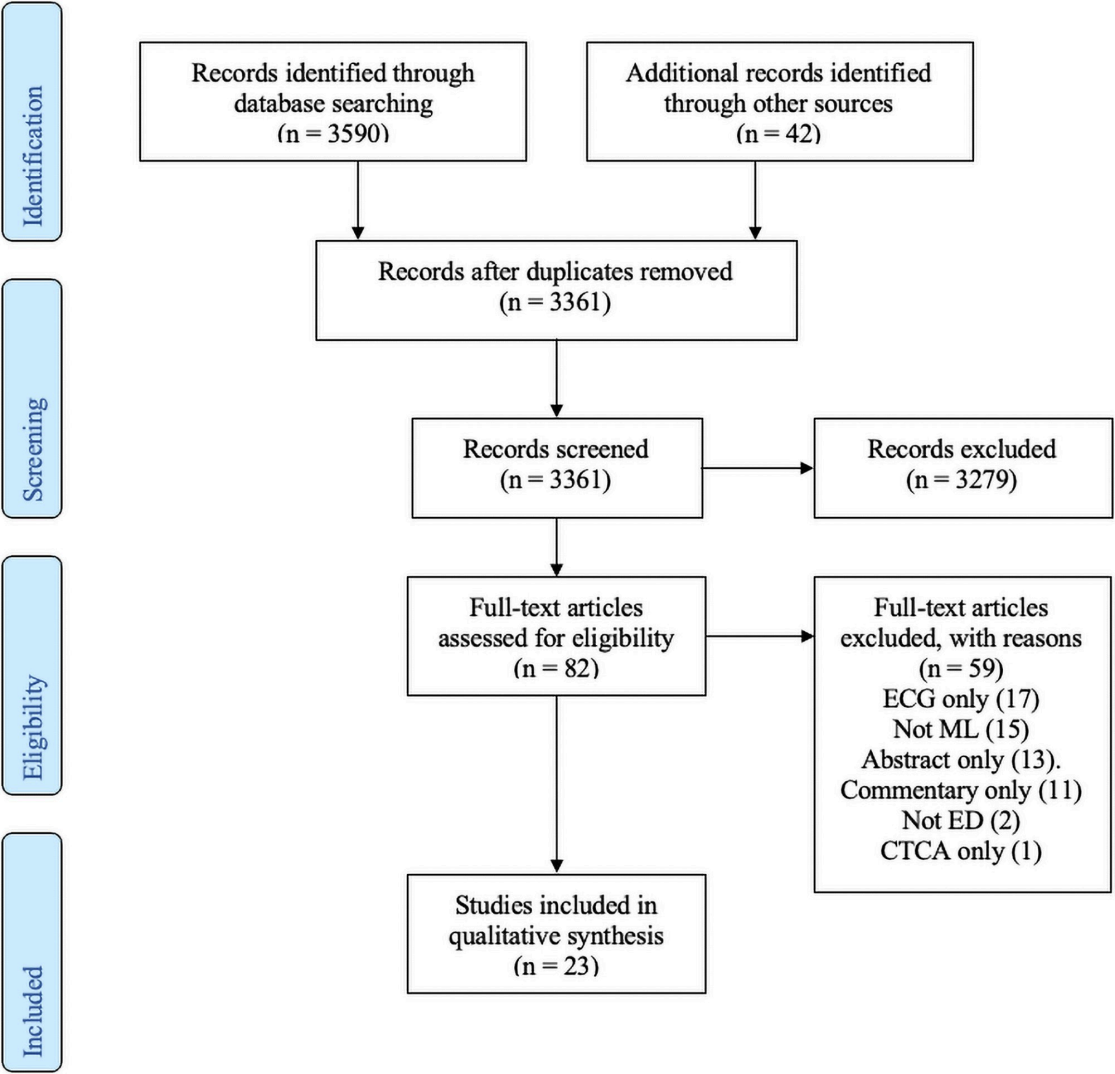


Following these exclusions, 23 studies remained for inclusion in our qualitative synthesis. There was no disagreement between the two reviewers as to study inclusion or results of data extraction. This process is summarised in a PRISMA Flow Diagram (Fig 1).

### Study characteristics

A summary of the included studies is shown in Table 1. There were 14 retrospective studies and 5 prospective studies. Four studies reported the development of a machine learning model retrospectively then tested it prospectively. The most common machine learning methods used were artificial neural networks (ANN) (14 studies), random forest (RF) (6 studies), support vector machine (SVM) (5 studies), and gradient boosting (2 studies).

**Table 1:** Summary of included studies.

Year	First author, reference	Study design	Primary Outcome	Single/Multi centre	Population	Data (Summary)	Number of input variables in model	ML Method	Comparison	Results of best ML model and of comparison (if applicable)
2020	Zhang et al. [31]	Retrospective, then prospective	30-day AMI 30-day all-cause mortality	Multicentre	85,254 patients with chest pain	Demographics, BMI, PMHx, Troponin, Labs	14	RF	LR, SVM, KNN	External validation AMI < 1 month Random forest AUC 0.907 All-cause Mortality <1 month Random forest AUC 0.888
2020	Wu et al. [32]	Retrospective	MACE within 90 days of ED presentation	Multicentre	938 patients with chest pain	Demographics, PMHx, ECG, Troponin, Labs,	5 in full 3 in reduced	ANN, RF, SVM	HEART Score	Internal validation Full Model AUC 0.853 Reduced Model AUC 0.808
2020	Mao et al. [33]	Retrospective	7-day MACE	Multicentre	833 patients with chest pain	Demographics, Sx, Exam, Troponin, Labs	Unknown	Gradient Boosting, SVM, LR	HEART Score	Internal validation XGBoost AUC 0.822 SVM AUC 0.649 LR AUC 0.667 HEART Score AUC 0.702
2019	Wu et al. [34]	Retrospective	Diagnosis of NSTEMI vs Angina	Single centre	268 patients with NSTEMI or unstable angina	PMHx, ECG, Troponin, Labs	9	ANN	None	Internal validation ANN AUC 0.962
2019	Than et al. [27]	Retrospective, then Prospective	Diagnosis of type 1 MI during the index admission	Multicentre	11011 ED patients presenting with suspected MI	Demographics, Troponin	4	Gradient Boosting	European Society of Cardiology (ESC) 1-hour and 3-hour algorithms.	External validation MI3 AUC 0.963 Sensitivity (low risk) 97.8% Specificity (high risk) 96.7% ESC 0/3-hour pathway Sensitivity (low risk) 82.5% Specificity (high risk) 92.2%
2017	Liu et al. [35]	Retrospective	72-Hour MACE.	Single centre	797 ED patients with chest pain	Demographics, PMHx, Vitals, ECG, Troponin	26	Extreme Learning Machine (ELM)	HEART score, TIMI score, GRACE score	Internal validation ELM AUC 0.778 Sensitivity 0.753 Specificity 0.704
2016	Berikol et al. [36]	Retrospective	Diagnosis of ACS	Single centre	228 patients with chest pain and a cardiology consult	Demographics, PMHx, ECG, Troponin, Labs, Echo	7	SVM, ANN, Naïve Bayes, LR	None	Internal validation SVM Sensitivity 98.22 Specificity 100
2014	Liu et al. [37]	Retrospective	30-day MACE	Single centre	648 patients with chest pain	ECG, Vitals	21	RF	TIMI	Internal validation ML Score AUC 0.81 TIMI AUC 0.71
2014	Liu et al. [38]	Prospective (observational)	3-day MACE	Single centre	702 patients with chest pain	Demographics, PMHx, Vitals, ECG	3 (most relevant) 23 (all)	RF, SVM	TIMI and MEWS	Internal validation Scoring system (3 variables) AUC 0.812 Scoring system (23 variables) AUC 0.736 TIMI AUC 0.637 MEWS AUC 0.622
2010	Ha et al. [39]	Retrospective	Diagnosis of AMI vs Angina vs other chest pain disease	Single centre	478 patients with chest pain	Demographics, Troponin, Labs	39	Decision tree	SVM, ANN	Internal validation C5.0 model Accuracy of 94.18%
2007	McCullough et al. [40]	Prospective	30-day MACE (by Gender)	Single centre	2148 patients with chest pain	Demographics, PMHx, Estrogen status (women only), Sx, physician assessment	14	ANN	None	Internal validation Female patients (all features) Trained on female data only AUC 0.8988 Trained on all data AUC 0.9037 Male patients (all features) Trained on male data only AUC 0.8793 Trained on all data AUC 0.8552
2006	Green et al. [41]	Retrospective	Diagnosis of ACS	Single centre	634 patients with chest pain and ECG	Demographics, PMHx, Meds, Sx, Vitals, ECG	38	ANN, LR	None	Internal validation ANN ensemble AUC 0.80
2005	Conforti et al. [42]	Retrospective	Diagnosis of AMI	Single centre	242 patients with chest pain	Demographics, Vitals, ECG, Troponin, Labs, Echo	105 and 25	SVM (Polynomial, Gaussian, Laplacian Kernels)	None	Internal validation Testing correctness 25 Variables—97.5% 105 Variables—97.5%
2005	Harrison et al. [43]	Retrospective, then prospective	Diagnosis of ACS	Multicentre	3147 patients with chest pain.	Demographics, PMHx, Sx, Exam, ECG	8 13 20 40	ANN	LR.	External validation 13-factor ANN Hospital 2—AUC 0.93 Hospital 3—AUC 0.95
2003	Hollander et al. [44]	Prospective	Change in admission to hospital and disposition following implementation of a previously developed ANN.	Single centre	4492 patients with chest pain before implementation 432 patients with chest pain after implementation	Demographics, PMHx, Sx, Exam, ECG, Troponin, Labs	40	ANN	Standard practice prior to implementation of neural network	External validation Admission rate Before implementation of ANN—62.7% After implementation of ANN—66.6% Patients for whom ANN output changed management during real time use– 2 (<1%)
2002	Baxt et al. [45]	Retrospective	Ischaemic (MI/UA/Angina) vs non-ischaemic chest pain	Single centre	2204 patients with chest pain	Demographics, PMHx, Exam, ECG, Troponin, Labs	40	ANN	ACI-TIPI, Goldmanm LR	Internal validation ANN Sensitivity 88.1% Specificity of 86.2%
2002	Baxt et al. [46]	Retrospective	Diagnosis of AMI	Single centre	2204 patients with chest pain and an ECG	Demographics, PMHx, Sx, Exam, Vitals, ECG, Troponin, Labs	40	ANN	LR	Internal validation ANN AUC 0.982 LR AUC 0.870
1998	Tsien et al. [47]	Retrospective	Diagnosis of AMI	Multi centre	1752 patients with chest pain	Demographics, PMHx, Sx, ECG	45	Decision tree	Goldman Decision Tree, LR equation	Internal validation Decision tree AUC 0.9404 Logistic regression AUC 0.9428
1998	Chazaro et al. [48]	Retrospective	Diagnosis of AMI	Single center	563 patients with chet pain	Demographics, PMHx, Sx, Meds	95	ANN	ED Physician LR	Internal validation ANN Sensitivity 0.85 Specificity of 0.91 ED physician Sensitivity 87% Specificity 78%) LR Sensitivity 8l% Specificity 86%
1997	Kennedy et al. [49]	Retrospective, then prospective	Diagnosis of AMI	Multicentre	Retrospective 290 patients with chest pain Prospective 86 patients with chest pain	Demographics, PMHx, Sx, Exam, ECG	53	ANN	Physician, Myoglobin (3 and 16hr)	External validation Physician Accuracy 65.1 ANN Accuracy 73.6 Myoglobin Accuracy 82.4
1996	Baxt et al. [50]	Prospective	Diagnosis of AMI within 3 days of ED presentation	Single centre	1070 patients with chest pain	Demographics, PMHx, Sx, Exam, ECG	20	ANN	Physician.	Internal validation Physician Sensitivity 73.3% Specificity 81.1% ANN Sensitivity 96.0% Specificity 96.0%
1991	Baxt. [51]	Prospective (Validation)	Diagnosis of AMI	Single centre	331 patients with chest pain	Demographics, PMHx, Sx, Exam, ECG	20	ANN	Physician	Validation study of previously derived model Physicians Sensitivity 0.777 Specificity 0.847 ANN Sensitivity 0.972 Specificity 0.962
1990	Baxt. [14]	Retrospective	Diagnosis of AMI	Single centre	356 patients with chest pain	Demographics, PMHx, Meds, Sx, Vitals, Exam, ECG	41	ANN	None	Internal validation ANN Sensitivity 92% Specificity 96%

Abbreviations

AMI—Acute Myocardial Infarction

ACS—Acute Coronary Syndrome

MACE—Major Adverse Cardiovascular Event

Demographics—Including age and gender

Sx—Patient’s symptoms

Exam—Clinical exam findings

PMHx—Past Medical History (including family history and smoking status)

Vitals—Patients vital signs, including heart rate, blood pressure, respiratory rate, oxygen saturation, and temperature

Labs—Blood tests (excluding troponin)

Meds—Patients medication

ECG—Electrocardiogram

Echo—Echocardiogram

RF—Random forest

SVM—support vector machine

KNN—k-nearest neighbours

XGBoost—eXtreme Gradient Boosting

ANN—Artificial neural network

AUC—Area Under Receiver Operating Characteristic Curve

LR—Logistic regression

HEART—Heart Score

TIMI—Thrombolysis In Myocardial Infarction Score

MEWS—Modified Early Warning Score

ACI-TIPI—Acute Cardiac Ischemia Time-Insensitive Predictive Instrument

The most common primary outcomes assessed were diagnosis of Acute Myocardial Infarction (AMI) (12 studies), and prognosis of major adverse cardiovascular event (MACE) (6 studies). Three studies looked at diagnosis of ACS (including unstable angina). The NSTEMI/STEMI paradigm formally replaced the Q-wave/Non-Q-wave MI paradigm in 2000 [52]. This review identified 17 studies published since 2000 of which 4 studies excluded STEMI patients and 13 did not.

Sixteen studies used data from a single site and 7 studies used data from multiple sites. The largest number of sites used was by Than et al, an international collaboration using 12 cohorts of patients [27]. They developed their model using training data from 2 cohorts then validated their models using prospectively collected data from 7 cohorts.

The population size assessed varied greatly. The largest population was 85 254 patients by Zhang et al., who used data collected from chest pain presentations to three hospitals between 2009 and 2018 [31]. The smallest population assessed was 228 patients by Berikol et al. [36] Fourteen studies used a population of under 1000, seven studies had a population of between 1001 and 10 000, and two studies used a population of over 10 000.

The most frequently used prediction variables were demographics (21 studies), past medical history including smoking status and family history (18 studies), and ECG result (17 studies). Troponin was used in 12 studies. Only one study (Than et al) used serial troponins [27].

Laboratory tests other than troponin were used in 10 studies. Patient symptoms were used in 12 studies and examination findings were used in 8 studies.

The number of predictor variables used in the ML models varied. The median number of input variables used in the ML models was 23. Overall, 5 studies used models with 10 or less input variables, 8 studies used models with 11–30, 7 studies used models with 31–50, and 2 studies used models with more than 50 input variables. The number of variables was unknown in one study and attempts to contact the study authors to clarify this were unsuccessful. The largest number of input variables was 95 by Chazaro et al. and the smallest number used by Than et al. who used only 4 variables (age, gender, paired high-sensitivity troponins, rate of change of high-sensitivity troponin) [27, 48]. Liu et al found that their 3 variable model produced better results than their complete 23 variable model in predicting 3-day MACE (AUC of 0.812 vs AUC of 0.736), concluding that “more predictors do not necessarily guarantee better prediction result” [37].

### Diagnosis of AMI or ACS

16 studies used ML algorithms to diagnose AMI or ACS in patients presenting to the ED with chest pain. Tsien et al. in 1998 and Harrison et al. in 2005 were the only authors to report that ML techniques did not outperform logistic regression and also suggested that appropriate models for use in clinical practice may be able to be developed with relatively few data items [43, 47]. Than et al. used ML to develop their “MI^3^ clinical support tool” which achieved a high AUC (0.963) in diagnosing type 1 myocardial infarction in the index admission when prospectively validated, and achieved similar performance in early and late presenters [27]. Their algorithm incorporated paired high-sensitivity troponins collected at presentation at another early, yet flexible time point. Their MI^3^ clinical support tool was designed to be used as a continuous measure but could also be adapted to work in the current paradigm of low/high risk chest pain. Using an example low risk threshold (69.5% of patients in their test set) they achieved a negative predictive value of 99.7% and sensitivity of 97.8%. At a high-risk threshold (10.6% of patients in their test set) they achieved a positive predictive value of 71.8% and specificity of 96.7%. At these thresholds their algorithm outperformed the European Society of Cardiology 0/3-hour pathway.

### Prognosis (prediction of MACE and mortality)

In total 7 studies used ML algorithms to predict the prognosis of patients presenting to the ED with chest pain. 6 studies looked at composite prognostic outcomes (MACE) and 1 study (Zhang et al 2020) looked separately at 30-day all-cause mortality and 30-day AMI following ED presentation [31]. Prognostication studies varied in the timeframes considered. The longest time frame assessed was 90-day MACE by Wu et al. [32] Wu et al. used ML to select features for their risk stratification model, developing a full model that contained invasive (blood tests) variables, and a reduced model that only contained non-invasive variables. They also identify that in their data, QTc prolongation was a potentially novel predictor of MACE. Their full model achieved an AUC of 0.853 and their reduced model achieved an AUC of 0.808.

The shortest timeframe considered was by Liu et al, who applied ML to select variables from 8 vital signs 15 heart rate variability parameters to build a model to predict 3-day MACE [37]. Their top performing model contained only 3 variables and achieved an AUC of 0.812, outperforming the TIMI score (AUC 0.637) and the modified early warning score (AUC 0.622). Applying an arbitrary low/high risk cut-off score gave a sensitivity of 82.8% and specificity of 63.4%. The variables required for the model could be quickly and obtained non-invasively through collection of routine vital signs and a 5-minute ECG. In a subsequent paper Liu et al. developed a ML score that again incorporated vital signs and ECG heart rate variability data to predict 30-day MACE. Their ML score achieved an AUC of 0.81, again outperforming the TIMI score (AUC 0.71) [38].

Zhang et al. used a ML algorithm based on demographic information, past medical history (PMHx), and laboratory tests to predict AMI and all-cause mortality within one month [31]. In prospective validation their RF model achieved an AUC of 0.907 for AMI < 1 month and an AUC of 0.888 for all-cause mortality < 1 month.

Than et al. conducted a pre-planned secondary analysis on their MI3 algorithm to assess ability to predict patients who suffered an MI in the 30-days following discharge [27]. Their MI3 algorithm achieved an AUC of 0.957, and setting (arbitrary) low/high risk threshold values gave a sensitivity of 96.6% and PPV of 71.9% respectively.

McCullough et al used ANN to predict 30-day MACE based on demographics, PMHx, estrogen status (women only), patient symptoms, and subjective physician initial assessment of the chest pain (assessed as either Typical cardiac pain,” “Atypical cardiac pain,” or “Probable non-cardiac pain.”) [40]. They developed prediction models for male and female patients. They found that adding the subjective physician assessment to their model improved the performance of the model more for male patients (average improvement of 5%), than for female patients (average improvement of 1.48%). When their model used all features available and was trained all available data (male and female) it achieved an AUC of 0.9037 for females and 0.8552 for males. Training the model on only male data improved the AUC for males to 0.87.

### Comparisons

The most frequently used comparator was logistic regression (6 studies). The HEART score was used as a comparator in 4 studies, the TIMI score was used as a comparator in 3 studies. Other comparators used included (ESC) 1-hour and 3-hour algorithms, the GRACE score, and MEWS. All existing chest pain risk stratification scores were outperformed by various ML models in all studies in which they were compared. Two studies compared the performance of various ML algorithms to one another. Zhang et al. compared RF, SVM, and KNN for AMI and mortality prognosis [31]. Ha et al. compared Decision tree, SVM, and ANN to diagnose MI [39]. In both cases decision trees (including RF) outperformed other ML algorithms. LR was used as a comparator in 6 studies. ML models outperformed LR in four studies. Tsien et al. and Harrison et al. reported that their ML models did not show increased performance when compared to logistic regression [43, 47]. Four studies compared ML to physician. All four studies compared an ANN to a physician in the diagnosis of AMI. Chazaro found the ED physician achieved greater sensitivity (87%) than the ANN (85%), however lower specificity (78% vs 91%) [48]. In the three other studies, the physician was outperformed by the ANN in all metrics [49–51]. Six studies did not include a comparator.

### Integration into practice

Only 2 studies reported integrating the ML model into clinical practice. In 2003 Hollander et al. evaluated consecutive ED patients with chest pain before and after the implementation of an ANN [44]. The treating emergency physicians were provided with real-time outputs of the neural network, which had previously achieved 95% and specificity of 96% in diagnosing acute myocardial infarction. The implementation of the neural network did not significantly change admission decisions. There were only 2 patients (<1%) for whom the neural network output altered the physician disposition decision during real-time use. In a follow-up survey, 70% of physicians believed the neural network to be correct, and 52% had confidence in the network output. However only 7% stated they used the network score in their decision making. The main reasons given for not using the neural network score in their decision making was that the data were “presented to late’ and that the results ‘confirmed clinical suspicion but did not alter it”.

In 2020 Zhang et al. retrospectively developed a ML model for predicting MACE in 85,254 patients with chest pain in the EDs of three hospitals [31]. They used 14 clinical variables previously suggested to predict MACE including demographics, PMHx (defined as diagnosis before the index visit), and high-sensitivity troponins. They found a RF model using an oversampling approach outperformed SVM, KNN, and LR. After one month of testing and validation, the ML model was launched in their Hospital Information System to assist ED physicians with decision-making in real time. Prospective validation of the AI prediction model by new patients showed AUCs of 0.907 for AMI within 1 month and 0.888 for all-cause mortality within 1 month. Their model was able to automatically and rapid capture the necessary variables (including high sensitivity troponin) from their EHR when the physician requested the ML prediction. The authors acknowledge that they did not assess the impact of the ML prediction model on clinical practice, and that the impact on emergency physician decision making, change in clinical practice, and patient outcomes may need by be evaluated in future work.

### Availability of code and dataset

Only 2 studies shared their datasets (Table 2). Conforti et al. provided publicly available link to their dataset, however this link no longer works [42]. Wu et al. stated that their dataset was available on reasonable request [32]. The code used for the ML models was not publicly available in any studies. The ML model used by Than et al. is proprietary but is available from the authors for research purposes on request [27]. Chazaro et al did not share their algorithm however did provide the numeric values for hyperparameters for their ANN that achieved their best results [48].

**Table 2:** Availability of dataset and code for included studies.

Study	Dataset publicly available	Code available
Zhang et al. [31]	No	No
Wu et al. [32]	Yes*	No
Mao et al. [33]	No	No
Wu et al. [34]	No	No
Than et al. [27]	No	Yes ^
Liu et al. [35]	No	No
Berikol et al. [36]	No	No
Liu et al. [37]	No	No
Liu et al. [38]	No	No
Ha et al. [39]	No	No
McCullough et al. [40]	No	No
Green et al. [41]	No	No
Conforti et al. [42]	Yes~	No
Harrison et al. [43]	No	No
Hollander et al. [44]	No	No
Baxt et al. [45]	No	No
Baxt et al. [46]	No	No
Tsien et al. [47]	No	No
Chazaro et al. [48]	No	Yes^#^
Kennedy et al. [49]	No	No
Baxt et al. [50]	No	No
Baxt. [51]	No	No
Baxt. [14]	No	No

* available on request.

^ Model proprietary but available on request for research purposes.

^~^ Link provided to publicly available dataset in the paper no longer works.

^#^ partially, configuration and settings provided in text table.

### Study quality—Risk of bias within and across studies

A summary of the PROBAST assessment is provided in Table 3. Overall, 16 studies were considered to have a high risk of bias and 4 low risk of bias. 5 studies had high applicability concerns and 7 studies had low applicability concerns. There were only 3 studies that were considered low risk of bias and had low applicability concerns. Only 4 studies externally validated their ML models. Only one study (Then et al) refers to a previously published or registered protocol [27]. All but two studies report positive results for machine learning algorithms, which raises the question if reporting bias may be present.

**Table 3:** PROBAST assessment of the included studies.

Study	Risk of Bias (ROB)				Applicability			Overall	
	Participants	Predictors	Outcome	Analysis	Participants	Predictors	Outcome	ROB	Applicability
Zhang et al. [31]	+	+	?	+	+	+	+	?	+
Wu et al. [32]	+	?	?	-	+	?	?	-	?
Mao et al. [33]	+	?	?	?	-	?	?	-	-
Wu et al. [34]	+	+	?	-	-	?	?	-	-
Than et al. [27]	+	+	+	+	+	+	+	+	+
Liu et al. [35]	+	+	?	-	+	+	?	-	?
Berikol et al. [36]	+	?	-	-	-	?	-	-	-
Liu et al. [37]	+	+	+	-	+	+	+	-	+
Liu et al. [38]	+	+	+	-	+	+	?	-	?
Ha et al. [39]	-	+	?	-	?	+	?	-	?
McCullough et al. [40]	+	+	?	-	+	+	?	-	?
Green et al. [41]	+	+	+	-	+	+	+	-	+
Conforti et al. [42]	-	-	-	-	+	+	?	-	?
Harrison et al. [43]	+	+	+	+	+	+	+	+	+
Hollander et al. [44]	+	+	+	+	+	+	+	+	+
Baxt et al. [45]	+	+	+	-	+	+	+	-	+
Baxt et al. [46]	+	+	+	-	+	?	+	-	?
Tsien et al. [47]	+	+	?	+	+	+	?	?	-
Chazaro et al. [48]	+	+	?	-	+	+	?	-	?
Kennedy et al. [49]	?	+	?	-	+	+	?	-	?
Baxt et al. [50]	+	?	+	+	+	+	?	?	?
Baxt. [51]	+	?	+	—	+	+	?	+	?
Baxt. [14]	+	?	?	-	+	+	-	-	-

PROBAST = Prediction model Risk Of Bias ASsessment Tool; ROB = risk of bias.

+ indicates low ROB/low concern regarding applicability.

− indicates high ROB/high concern regarding applicability; and? indicates unclear ROB/unclear concern regarding applicability.

## Discussion

### Interest and early work

This systematic review has found that there has been long-standing interest in the applications of ML to undifferentiated chest pain the ED, and that ML techniques have achieved impressive results both diagnostic and prognostic applications. These results could potentially relieve emergency physicians of diagnostic burden, deliver improved care to patients, and assist the health systems to provide care with greater efficiency. Over the last decade there has been rapid growth in technological capability, digitalisation of information, and dataset size. ML has become increasingly powerful while also becoming more accessible. Models described by Baxt in 1990 that took up to 48 hours to train would train in seconds today [14].

### Compared to physicians and current standard of care

Pioneering work by Baxt in the 1990s found that “the non-linear artificial neural network performs more accurately than either physicians or other computer-based paradigms” [53]. Despite this, relatively few studies compared ML to physicians, and no study since 1998 has directly compared ML to physicians for the diagnosis or prognosis of undifferentiated chest pain in the ED. More recent studies that included comparisons have instead compared ML to current risk stratification tools such as the TIMI and HEART score. Though routinely used in clinical practice, there is emerging evidence that the HEART score may not perform better than clinical gestalt in certain clinical scenarios [54]. As ML tools become integrated into practice it will continue to be important to compare ML tools to physicians.

### Small datasets

ML model performance tends to improve as dataset and model size increases [55]. Large high quality clinical datasets are difficult to obtain and their size is limited by the number of patient presentations. There is a trend to supplement real datasets with synthetically realistic generated data. This allows for arbitrarily large datasets with corresponding improved model performance. Class imbalance is also a common problem, with some data classes being abundant and other classes such as mortality expectedly being rare. New DL techniques have been developed to address this problem [56].

Reported ML architectures used by the studies in this review remain small compared to state-of-the-art architectures used in other fields, and the vast majority of datasets used were very small by modern machine learning standards. For perspective, State-of-the-art computer vision models are often trained on a dataset containing more than 14 million images [20]. A recently developed natural language processing algorithm (GTP-3) uses 499 billion tokens as input to train [22]. Rajkomar et al. predicted mortality through training on a dataset containing over 216,000 patients and over 46 billion data points [25]. At these scales, training cost becomes a significant consideration and prohibitively expensive to many researchers. Though training large models may be slow and expensive, after training predictions can be delivered rapidly using much less computational power, such as found in standard computers or mobile telephone. Zhang et al. reported that the time taken to generate prediction results following the ED physician clicking the appropriate button was < 1 second [31]. There is potential that large models could be developed and trained by researchers with the appropriate resources, then if these models are publicly available, they can be adapted to and validated on local data, reducing training time and cost. This may be especially important in low-resource settings.

### Omitted data categories

Multiple studies achieved impressive results, despite not including some data routinely used by emergency physicians in the evaluation of undifferentiated chest pain. Almost half (11/23) of the studies assessed did not take into account patients’ symptoms. Incorporating unstructured data in datasets remained a challenge. Interpretations of echocardiogram and ECG data were used in all datasets that included them. No studies used deep learning to incorporate unstructured image or ECG data, and no studies applied natural language processing to incorporate free text clinical notes. Interestingly no studies incorporated chest x-rays, though they are routinely used in the work up of undifferentiated chest pain in the ED.

McCullough et al. conducted the only study that included emergency physician impression as an input in a ML algorithm [40]. It is perhaps reassuring that including the emergency physician impression improved their models results, however interestingly the results were improved more for male than female patients. Previous work has suggested male and female patients with chest pain may be treated differently [57]. It is unknown if their result is a reflection of this disparity. Their model achieved great results for female patients without the inclusion of emergency physician assessment. It is interesting to consider where the emergency physician is left if future studies find they are outperformed by an ML model, and the inclusion of their subjective assessment is not found to improve the model. The future role of the emergency physician may move from diagnosis of undifferentiated cases to interpreting and communicating results to patient and participating in shared decision making. It unlikely that ML models will be able to encroach on the emergency physician’s many other roles including resuscitation, practical skills, and team management.

### Predictor variables

As is common in ML research, multiple studies experimented with different numbers of input variables and found that more variables did not necessarily improver results, or the addition of more variables only marginally improved performance. Liu et al astutely suggested that a simple model using non-invasive variables could play a role in patient triage [37]. ML also showed potential to identify and incorporate novel risk variables such as heart rate variability parameters and correct QT interval in ECG [34]. Troponin is an important component of the universal definition of MI [3]. The study cohorts were patients presenting with a symptom of myocardial ischemia (chest pain), and so all those with a rise and/or fall of troponin values (with at least 1 value above the 99th percentile) will meet current definitions for MI. Including a variable used in the definition of MI as an input in a ML model to predict MI is problematic and will likely lead to optimistic estimates of model performance [30]. In many cases, initial troponin measurements are likely to have formed part of the information used to determine the outcome.

### Human interpretability

Though there are differing opinions, it is generally accepted that ML model output will need to be interpretable to be accepted and used in the health care setting [58]. There is now considerable research focusing on developing “explainable AI” [59]. No studies provided a human interpretable output of the diagnostic reasoning of their algorithms alongside their output. Than et al. developed an app mock-up that make the results human understandable [27]. This is an important step in communication of results but does not provide glimpse into the ‘black box’ that is the algorithm. Given the size, complexity and level of abstraction of the underlying models, interpretation is generally infeasible [24]. It may not be possible to achieve any more than an illusion of understanding. However, emergency physicians routinely prescribe medication with unclear mechanisms of action, but for which there is robust safety and efficacy data [60]. If a ML model consistently demonstrates predictable accuracy and safety in a wide variety of circumstances, it may be accepted despite remaining a ‘black box’.

### Human factors affecting model implementation

Few studies considered the human factors that are involved in the implementation of ML algorithms into practice. Hollander et al. provided an important singular example of a study that evaluated the effect of algorithm implantation on clinical decision making, showing that despite implementing an ANN that was previously reported to outperform clinicians, few used it and it did not change clinical practice. New ML based diagnostic and prognostic technologies may be rejected by emergency physicians, especially if the results are not timely and do not change management [44]. Physicians are likely to remain skeptical of an unexplainable black box. There has also been no evaluation of ED patients attitudes and opinions on the use of ML in their care. To achieve physician and patient acceptance of ML technologies will likely require deep consideration of the human factors involved.

### Ethical and legal issues

Zhang et al. point out that the implementation of ML prediction models in healthcare raises ethical and legal issues, including malpractice liability for both technology manufacturers and emergency physicians [31]. There is justified concern that important decisions could be based on output of an algorithm that isn’t or fundamentally can’t be understood by a human [61]. Current legal doctrine is likely to be inadequate to address ML-related medical malpractice [62].

### Sensitivity and specificity

Physician, patient, and institutional risk tolerances differ. Achieving higher sensitivity at the expense of lower specificity will lead to more false positives, and the resulting over investigation of these cases can paradoxically cause more harm than if the test wasn’t conducted. The concept of ‘test-threshold’ shows the point at which risks of harm from false positive tests are equal to the risks of not testing [63]. Patients with risk below the test threshold do not benefit from further testing. This leads to a mathematically optimal miss rate. Kline et al. estimated that attempting to achieve a miss rate of under 2% for investigating patients with suspected cardiac chest pain may cause more harm through over investigation [64]. This miss rate may not be the miss-rate that clinicians are comfortable adopting, and physicians may be doing more harm than good by adopting unrealistically low miss rates for low-risk patients presenting with chest pain [65]. It remains to be seen if ML can solve this dilemma.

### Implementation

Despite over 30 years of promising results, integration of ML algorithms into widespread clinical practice is yet to occur. Heterogeneity amongst healthcare systems is likely a significant barrier. Zhang et al. were able to deploy their model into practice, but also note that while providing a proof of concept, the model may not be generalisable to other hospitals [31]. They suggest that re-training and testing in other hospitals could overcome this issue. A mock-up app developed by Than et al. shows thoughtful consideration of how a centralised ML algorithm could be used in a low-resource setting, and how the results may be presented through a phone application to both physicians (diagnostic metrics) and patients (graphical format) [27]. Implementation of ML algorithms will require health system monitoring, oversight and development of algorithm stewardship frameworks to ensure that algorithms are used safely, effectively, and fairly in diverse patient populations [66].

### ML reproducibility crisis, algorithm ownership

Reproducibility is a foundation of the scientific method. There is growing recognition that ML research is suffering from a reproducibility crisis [67]. This review found that few studies publicly shared their code or dataset. Furthermore, methodological details were insufficiently documented to allow for replication in many studies. Recent medical ML studies have been criticised for lacking sufficiently detailed methods, and not sharing data, algorithm code, or details of the computational environment that generated the published findings [68]. Sharing of data and code is widely viewed as important, and the lack of such sharing undermines the scientific value of the research [68]. Previously identified barrier to transparent and reproducible ML research include the privacy and ethical implications of sharing patient data, and the economic disincentives of sharing proprietary models [69].

Despite facing similar privacy challenges, the biomedical literature has shown some improvements in certain key indicators of reproducibility and transparency, and clear, detailed, and enforced guidelines have allowed for genomics researchers to share complex computational pipelines and sensitive datasets [68–71]. Solutions may involve creating a research culture that favours openness and replication, demonstration of the model on public datasets, or the ability of independent investigators able to access the data and verify the analysis prior to publication [68]. No studies identified by this review were replication studies. Ongoing effort to manage the tension between patient privacy, open science, and private enterprise is required.

### Future direction

There have not been any randomised clinical trials comparing a ML algorithm to physicians or current risk scoring tools for the risk stratification of chest pain. No studies have have evaluated for a change in patient orientated outcomes following the implementation of a ML algorithm into clinical practice. It remains essential to assess the impact these tools have on clinical decision making. ML algorithms have potential to both decrease or increase bias and any future implementation of such must be conscious of this and develop appropriate algorithm stewardship frameworks [66]. There is significant scope to incorporate further input variables into machine learning models including physician assessment, free text clinical notes, raw ECG data, point of care echocardiogram, and chest x-ray. There will likely be an increasing emphasis on model explainability, though it should be remembered that this may only give the illusion of understanding through abstraction of the underlying complexity. Despite using broad search terms such as “Chest Pain”, all studies included in this review focused only on MI/ACS and MACE. No studies attempted to diagnose other life-threatening cause of undifferentiated chest pain (such as pulmonary embolism or aortic dissection). Future research may attempt to broaden the scope of ML in undifferentiated chest pain.

Patients with acute coronary artery occlusion benefit from emergent reperfusion therapy [72]. Currently these patients are mainly identified by the presence of ST-elevation on ECG. There is a subset of patients with acute coronary artery occlusion who are not identified by the STEMI/NSTEMI paradigm [72]. While some studies used angiogram results as part of their outcome definition, no studies have attempted to identify patients with acute coronary artery occlusion. Future studies may use ML to attempt to identify patients who have acute coronary artery occlusion but who do not meet current STEMI criteria.

## Limitations

### Limitations—Study level

There are a number of limitations to this review. The majority (87%) of included studies were assessed to have either a high risk of bias, or high applicability concerns, and their results may not be generalisable to other settings. The majority of studies were also single centre, retrospective, and without prospective or external validation. The definition of MI and biomarkers used to define MI has also changed over time. The extended timeframe of this review means that many studies were done before the introduction of high sensitivity troponins and so results from earlier studies may not be applicable to the modern setting. Since being introduced into the definition of MI in 2000, few studies (4/17) excluded patients with STEMI. The clinical usefulness and applicability of ML scores to patients with STEMI is likely very low as they are often quickly identified on the basis of ECG alone, and there are well established existing treatment pathways for these patients (emergency reperfusion). There was inconsistent reporting of methods and results among studies. ML reporting guidelines are not well established or adhered to, though efforts are ongoing to change this [73–75].

### Limitations—Review level

Publication bias is known to be widespread in the medical literature. While there is no empirical evidence that it is present ML research, it is likely to be present, as in other fields of research. All but two study reported positive results for machine learning. Despite significant effort to develop broad and relevant search terms, some relevant research may be published under terms not included in the search. The search strategy also excluded abstracts, non-English articles. Quantitate synthesis was not performed due to a high level of study heterogeneity. Although this was expected and outlined in the research protocol, it means that this review does not provide a high level of evidence for the use of ML in undifferentiated chest pain. Machine learning is an evolving concept without a precise and universally accepted definition. Some definitions of ML include logistic regression, however, following common usage, this review did not consider it a ML technique.

## Conclusion

Research on applications of ML for undifferentiated chest pain in the ED has been ongoing for decades. ML has been reported to outperform emergency physicians and current risk stratification tools to diagnose AMI and predict MACE but has rarely been integrated into practice. Many studies assessing the use of ML in undifferentiated chest pain in the ED have a high risk of bias. It is important that future studies make use of recently developed standardised ML reporting guidelines, register their protocols, and share their datasets and code. Future work is required to assess the impact of ML model implementation on clinical decision making, patient orientated outcomes, and patient and physician acceptability.

## Supporting information

S1 Appendix Medline search strategy. (DOCX)

S1 Checklist PRISMA 2009 checklist. (PDF)
