# Supplementary material for: Applications of machine learning to undifferentiated chest pain in the emergency department: A systematic review
Source: PLoS One. 2021 Aug 24;16(8):e0252612. doi: 10.1371/journal.pone.0252612 (PMC8384172; doi:10.1371/journal.pone.0252612)
Supplement: S1 Appendix — (DOCX) [file pone.0252612.s001.docx]

**S1 Appendix – Medline Search Strategy**

| 1 | exp Artificial Intelligence/ |
| --- | --- |
| 2 | "ai".tw. |
| 3 | "expert system*".tw. |
| 4 | "reinforcement learning".tw. |
| 5 | "belief network*".tw. |
| 6 | "genetic algorithm*".tw. |
| 7 | "evolutionary algorithm*".tw. |
| 8 | "gaussian mixture mode*".tw. |
| 9 | "adaboost".tw. |
| 10 | "gradient boost*".tw. |
| 11 | "XGBoost".tw. |
| 12 | "multiple kernel learning".tw. |
| 13 | "relevance vector machine".tw. |
| 14 | "iterative learning".tw. |
| 15 | "discriminant analysis".tw. |
| 16 | "principle component analysis".tw. |
| 17 | "monte carlo method".tw. |
| 18 | "monte carlo method".tw. |
| 19 | "bayes theorem".tw. |
| 20 | "bayesian".tw. |
| 21 | "naive bayes".tw. |
| 22 | "decision tree*".tw. |
| 23 | "decision tree*".tw. |
| 24 | "fuzzy logic".tw. |
| 25 | "cluster analysis".tw. |
| 26 | "random forest".tw. |
| 27 | "supervised machine learning".tw. |
| 28 | "unsupervised machine learning".tw. |
| 29 | "computational intelligence".tw. |
| 30 | "computer reasoning".tw. |
| 31 | "deep learning".tw. |
| 32 | "machine learning".tw. |
| 33 | "natural language processing".tw. |
| 34 | "nlp".tw. |
| 35 | exp Neural Networks, Computer/ |
| 36 | "neural network*".tw. |
| 37 | "computer neural network*".tw. |
| 38 | exp Pattern Recognition, Automated/ |
| 39 | "automated pattern recognition".tw. |
| 40 | exp Diagnosis, Computer-Assisted/ |
| 41 | "computer?aided diagnosis".tw. |
| 42 | "computer?assisted diagnosis".tw. |
| 43 | exp Chest Pain/ |
| 44 | "chest pain".tw. |
| 45 | "angina".tw. |
| 46 | "myocardial infarc*".tw. |
| 47 | "STEMI".tw. |
| 48 | "NSTEMI".tw. |
| 49 | "myocardial ischaemia".tw. |
| 50 | "acute coronary syndrome*".tw. |
| 51 | "ACS".tw. |
| 52 | "MACE".tw. |
| 53 | "major adverse cardiac event*".tw. |
| 54 | exp Emergency Service, Hospital/ |
| 55 | "emergency department*".tw. |
| 56 | "emergency room*".tw. |
| 57 | "accident and emergency department*".tw. |
| 58 | "emergency hospital Service*".tw. |
| 59 | "emergency outpatient unit".tw. |
| 60 | "emergency unit*".tw. |
| 61 | "emergency ward*".tw. |
| 62 | "emergency Service*".tw. |
| 63 | "emergency medicine".tw. |
| 64 | "ED".tw. |
| 65 | exp Emergency Medicine/ |
| 66 | "emergency medicine".tw. |
| 67 | 1 or 2 or 3 or 4 or 5 or 6 or 7 or 8 or 9 or 10 or 11 or 12 or 13 or 14 or 15 or 16 or 17 or 18 or 19 or 20 or 21 or 22 or 23 or 24 or 25 or 26 or 27 or 28 or 29 or 30 or 31 or 32 or 33 or 34 or 35 or 36 or 37 or 38 or 39 or 40 or 41 or 42 |
| 68 | 43 or 44 or 45 or 46 or 47 or 48 or 49 or 50 or 51 or 52 or 53 |
| 69 | 54 or 55 or 56 or 57 or 58 or 59 or 60 or 61 or 62 or 63 or 64 or 65 or 66 |
| 70 | 67 and 68 and 69 |
